# Biographical—Dr. M. S. Dean

**Published:** 1882-03

**Authors:** 


### BIOGRAPHICAL.—DR. M. S. DEAN.

At a meeting of the Chicago Dental Society, the following report of the committee, appointed to prepare a biographical sketch and obituary notice in reference to the death of Dr. M. S. Dean, was unanimously adopted.

Dr. M. S. Dean was born in Pittsfield, Vermont, being the second of five children. His father was a minister of the Methodist church. His oldest brother was a physician and surgeon of repute at Milan, Ohio, who died in the service of his country in 1862, he being at that time a surgeon in the army.

The brother next younger than our deceased friend was also a physician at Milan, where he died just after the eldest brother's decease. The Rev. James A. Dean, A.M., D.D., now at the head of an educational institution at Durham, N. C., is the only survivor of the family. The subject of our sketch obtained the foundation of his education in the higher academies of his native State, and early commenced teaching.

In 1843, he studied medicine at Ogdensburg, N. Y., but afterwards determined to follow our specialty; in pursuance of which intention he studied dentistry with D. C. Ambler, M.D.

The commencement of his practice was made in Dundas, Gault, and Guelph, in Canada, from whence he moved to Milan, Ohio, the residence of his oldest brother. Here he remained in practice till 1852, when he went to Marshall, Michigan, establishing for himself a high reputation as a practitioner. In 1864, he made Chicago his home, where he remained until his untimely death.

Our friend was possessed of rare qualities of heart and mind. His nature was gentle, loving and sensitive, but he was as firm as a rock where principle was concerned.

Retiring and modest in his disposition, he yet won for himself hosts of friends wherever he was well known. The evidences of affectionate remembrances in which he was held by the friends of his earlier manhood, which one of your committee recently witnessed at his grave, attest to the remarkable affection he seems always to have attracted to himself.

As a practitioner he was conscientious to the last degree, and was thoroughly familiar with the theory of his profession; while as a diligent student his labor was chiefly directed toward original investigation, the results of which have aided materially in the unfolding of truths heretofore imperfectly seen.

He was not bound down by the mere affirmations of writers, no matter how eminent they might be, yet never presumptuous with his views, he only combated those of others when he felt convinced in his own mind that they were wrong. Such characteristics evince an earnest and unselfish devotion to his chosen calling, which was the most conspicuous feature of his life.

Aside from his professional attainments, he was possessed of the high degree of culture befitting the true professional gentleman.

Of the influence for good which he has exerted on our profession many of you know.

He was one of the earlier members of this Society, and his wise and moderate counsels more than anything else enabled us to tide over the period of infancy as a Society, and to avoid the rocks of contention and strife which have wrecked so many kindred organizations, or materially impaired their usefulness.

One of the founders of the Illinois State Dental Society, the same wise and prudent thought governed him in his connection therewith, and it may as justly be said of him in that connection, as in connection with this Society, that to him, more than to any other, is due the high position which that Society now holds.

He was for six years the Recording Secretary of the American Dental Association, and up to the time of his death was upon its Publication Committee, and for the admirable character of its published transactions we are mainly indebted to his able and untiring work. Twice has he been President of this Society, once of the Illinois State Dental Society, and once of the American Dental Association.

Whatever of outward honor has been in the power of the various organizations with which he was connected to bestow, has been freely given, unsought for by himself, while the universal respect and esteem felt for him, both in professional and social life, attest to the genuineness of the qualities that inspired them.

As a writer, he was careful and conscientious, not seeking notoriety, but always aiming to write to some purpose, and writing in the hope of aiding in the discovery of the truth. There are few men who would be so generally missed from the profession throughout the country or more sincerely mourned, while to us with whom he was more nearly associated his loss is irreparable.

It may truly be said of him, as was written by a professional brother from a neighboring State, "We owe this tribute to his memory: That he dignified and ennobled the profession of his choice."

In closing this brief record of the life of our dead friend, we beg to lay upon the altar of his memory the accompanying obituary:

Death has entered our ranks with so sudden and terrible a shock that we are stricken almost dumb.

He has laid his hand upon the one--the best beloved, the most honored, the most respected--and our hearts are bowed down with a grief we cannot find words to express.

In the life of our friend we may read the history of a lofty ambition never prostituted to selfish ends, but pursued for the advancement of his chosen profession and the benefit of his fellow men; and while we cannot understand the decree that took him from us in the height of his usefulness, we are grateful for the tenderness which tempered the blow, and permitted his life to close so peacefully, without one sigh of pain.

He is gone! The ripe scholar, the true gentleman, the wise counsellor, the generous friend! We can never again feel the warm grasp of his friendship; we can no more listen to his words of counsel, nor will his genial smile again brighten our gatherings!

We MUST mourn, and while we strive to express our sorrow, any words we may utter can be but a feeble expression of what we feel; yet in our hearts he will have a monument which shall endure as long as memory shall last, and the example of his pure life and lofty devotion to all that was most worthy will ever be an incentive to us to follow the path in which he so bravely led the way.

